# Radiological and functional outcome in extra-articular fractures of lower end radius treated conservatively with respect to its position of immobilization

**DOI:** 10.4103/0019-5413.40258

**Published:** 2008

**Authors:** Sunil Rajan, Saurabh Jain, A Ray, P Bhargava

**Affiliations:** Department of Orthopedics and Traumatology, MGM Medical College and M.Y. Hospital, Indore, Madhya Pradesh, India

**Keywords:** Cast immobilization, dorsiflexion, extra-articular fracture of lower end radius, palmar flexion

## Abstract

**Background:**

Extra-articular fractures of lower end radius are conventionally immobilized in palmar flexion and ulnar deviation. In view of poor functional results, the conventional method of immobilization is giving way to dorsiflexed-immobilized method. The aim of our study is to evaluate and compare the radiological and functional outcome in extra-articular fractures of lower end radius treated conservatively with respect to its position of immobilization.

**Materials and Methods:**

Sixty-four patients, all above 20 years of age with closed extra-articular fractures of lower end radius who were treated conservatively by close reduction and below elbow cast application constitute the clinical material. Irrespective of fracture geometry the patients were randomly allocated to dorsal or palmar flexed immobilized position of wrist. Patients were followed up for a minimum six-month period. The radial tilt, palmar tilt and ulnar variance are measured at prereduction, postreduction and at 6 month followup. The results were scored by Demerit Scoring System of Saito.

**Results:**

All fractures united. Individual movement of dorsiflexion, palmar flexion, supination, pronation and radial-ulnar deviation were all significantly better in the dorsiflexed-immobilized group as compared with the palmar flexed immobilized group. Grip strength recovery with subjective assessment was better in the dorsiflexed group (77%) as compared to the palmar flexed group (23%). Radiological parameters were markedly better in the dorsiflexed group. Ninety-one per cent of patients in the dorsiflexed group had excellent to good results as compared to 66% in the palmar flexed group.

**Conclusion:**

Functional results of extra-articular fractures of lower end radius are superior if the fractures after reduction are immobilized in dorsiflexion of wrist rather than in conventional palmar flexion position.

## INTRODUCTION

Fractures of lower end radius give almost uniformly good result.^1^–^5^ Numerous previous studies, have taken the amount of displacement into consideration but very few have dwelt on the role of the position of immobilization as a parameter for comparing radiological and functional outcome.^1^,^6^–^9^ The present study was undertaken to evaluate the functional and radiolological outcome of conservatively treated extra-articular fractures when wrist was immobilized in dorsiflexion (DF) compared to immobilization in palmar flexion (PF).

## MATERIALS AND METHODS

Prospective study of ninety two patients in the age group of 20-60 years with closed extraarticular fractures of the lower end radius (i.e., all type A2 facture including types A-2.1, A-2.2 and A-2.3 along with A-3.1 and A-3.2 fractures as per AO classification) was conducted from September 2004 to July 2006. Standard anteroposterior (AP) and lateral radiographs of injured wrist were taken.^10^–^13^

Twenty-four patients were treated surgically while four were lost to follow-up. Thus 28 patients were excluded from the study. The remaining 64 patients formed the study group, out of which 45 patients had dorsal displacement and 19 patients presented with volar displacement and all were treated initially by below elbow plaster of Paris (POP) slab for a period of approximately five days followed by closed reduction and below elbow cast application under general anesthesia.^10^,^14^

Reduction of fractures was done under image intensifier guidance using appropriate reduction maneuver. Dorsal bending type fractures (Colles) having increased dorsal angulations, shortening and radial deviation of distal fragment were reduced by applying longitudinal traction, ulnar deviation and palmar flexion at fracture site. Similarly palmar bending fractures (Smiths) having a reverse deformity of palmar angulations, shortening and radial deviation were reduced by producing opposite deformity by giving longitudinal traction, ulnar deviation and extension at fracture site.

Once the fracture was reduced as seen under C-arm, the patients were allocated dorsal or palmar flexed attitude of the wrist alternately, irrespective of the fracture geometry and immobilized with a below elbow POP cast. Four patients in the DF group were excluded from the study as they were lost to follow-up. The degree of immobilization was either 15° PF or 15° DF. Extra-articular fractures with extreme displacement or grossly comminuted fractures that were not amenable to reduction by manipulation were treated surgically and not included in the study.

Active finger movements were taught during period of cast immobilization. Plaster removal was done at four weeks. It was followed by active exercises during the first week and following active and passive exercises one week later. During the first two weeks of cast removal a crepe support was given.

Assessment of pain, disability, i.e. limitation of motion, subjective evaluation was done.^8^ Radiological parameters, radial tilt, palmar tilt and ulnar variance were measured immediately post reduction, at 1 month (at cast removal), 3 months and 6 months to know the residual deformity. The radial tilt was measured as the angle between the distal radial articular surface on AP view to a line perpendicular to the long axis of the radius^11^–^13^,^15^,^16^ (normal = 22-23° range 13-30°). On lateral view the angle created between the articular surface of the distal radius and a line perpendicular to the long axis of the radius denoted the palmar tilt^11^,^13^,^15^,^16^ (normal = 11-12° range - 0-28°). The ulnar variance refers to the vertical distance between a line parallel to the proximal surface of the lunate facet of the distal radius and a line parallel to the articular surface of the ulnar head on AP view^11^–^13^,^15^,^16^ (normal = 2mm). Movements were measured in degrees from neutral position^8^ with the help of goniometer. Grip strength was measured as mm of Hg with the help of a dynamometer. The results were scored by Demerit Scoring System of Saito^8^ (Table 1) and by taking AP and lateral radiographs.

**Table 1:** Demerit scoring system of Saito^8^

I. Subjective evaluation		
Excellent	No pain, no disability, no limitation of motion	0
Good	Occasional pain, no disability, slight limitation of motion	2
Fair	Occasional pain, no particular disability if careful, some limitation of motion, feeling of weakness in wrist, activities slightly restricted	4
Poor	Pain, disability, limitation of motion, Activities markedly restricted	6
II. Objective evaluation		
a. Residual deformity		
Ulnar variance	0 ± 2 mm 1	1
Palmar tilt	11 ± 10 degrees	1
Radial tilt	23 ± 10 degrees	1
b. Range of movements		
Dorsiflexion	<45 degree	1
Palmar flexion	<30 degree	1
Ulnar flexion	<15 degree	1
Radial flexion	<15 degree	1
Supination	<50 degree	1
Pronation	<50 degree	1
c. Grip power		
Dominant	<½ Power of the opposite hand	1
	<2/3 Power of the opposite hand	2
Non-dominant	<½ Power of the opposite hand	1
	<2/3 Power of the opposite hand	2
d. Arthritic changes		
None		0
Minimal	Irregularity of articular surface sharpening of articular margin	1
Moderate	Narrowed joint space, osteophytes	2
Severe	Marked osteophytes, ankylosis	3
e. Complications		
Nerve complication		1-2
Stiff fingers		1-2
Ruptures tendons		1-2

The functional results of both groups using the above (Saito's) scoring system were calculated by adding all the points and were finally graded as follows:

Excellent 0-3, Good 4-9, Fair 10-15 and Poor 16-26. Both the DF group and PF group were compared with each other on the above mentioned parameters of Saito.

## RESULTS

The results are evaluated for 64 patients. Out of the remaining 64, 28 were males whereas 36 were females. The ratio of involved limb being dominant to non-dominant was 1:1.46. Out of 45 fractures displaced dorsally on pre-reduction X-ray, 25 were immobilized in 15-° DF attitude while 20 in PF. The other 19 has volar displacement on pre-reduction; following reduction nine were immobilized in 15-° DF attitude while 10 in PF. Thus the total number of patients immobilized in PF after reduction was 30 against 34 patients immobilized in DF.

### Subjective evaluation

Subjective evaluation^8^ was done on the basis of pain, restriction of movements and disability. At final follow-up 31 patients (91.17%) of DF immobilized group had excellent to good results as compared to 20 patients (66.66%) in PF immobilized group (*P* value = 0.013).

### Objective evaluation

#### Residual deformity

##### Radial tilt:^8^

**Figure 1A:**
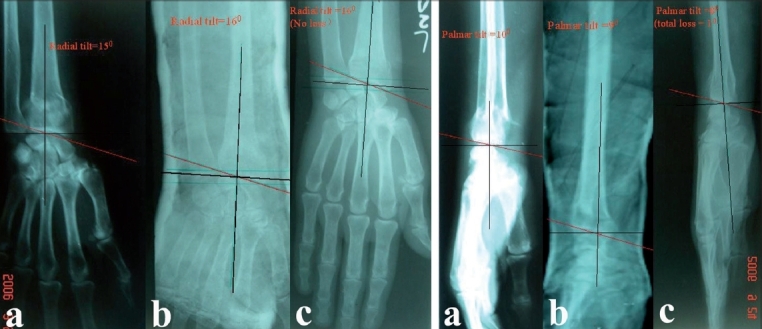
Pre-reduction AP x-ray (a) left wrist in dorsiflexion group shows fracture left radius with radial tilt of 15 degree. The radial tilt was corrected to 16 degree in postoperative xray (b) 6 months followup the xray (c) shows radial tilt of 16 degree. Overall no loss of radial tilt at 6 months followup. Fig. 1B - Pre-reduction lateral x-ray (a) of the same patient in dorsiflexion group shows palmar tilt of 10 degree which was corrected to 9 degree in postoperative xray (b) 6 months followup xray (c) shows palmar tilt of 8 degree. Overall 1 degree lost at 6 months followup

**Figure 2A:**
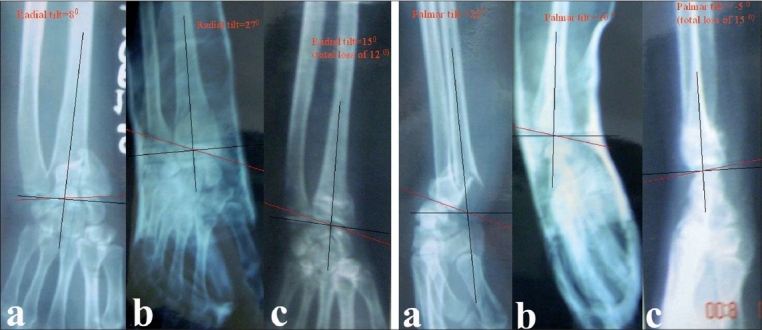
Pre-reduction AP x-ray of right wrist in (a) palmar flexion group shows fracture left distal radius with radial tilt of 8 degree. The radial tilt was corrected to 27 degree in postoperative xray (b) 6 months followup xray (c) shows radial tilt of 15 degree. Overall 12 degree loss of radial tilt at 6 months followup from post reduction xray. Fig. 2B - Pre-reduction lateral x-ray of the same patient in dorsiflexion group shows palmar tilt of 21 degree. It was corrected to 10 degree in postoperative xray (b) 6 months followup xray (c) shows palmar tilt of 5 degree. Overall 5 degree lost at 6 months followup

Successive follow-up showed decrease in tilt in both groups. At final follow-up 25 patients (73.52%) of DF group had 13 to 33° radial tilt as compared to 14 patients (46.7%) in PF group (*P* value = 0.002561). In both groups all patients showed radial tilt with 13-33° in immediate post reduction period. At one month follow-up one in the DF group and five in the PF group had lost correction of radial till. On subsequent follow-up at three and six months, four and seven respectively lost correction as compared to 0 and 5 in DF group Table 2, Fig. 1A and Fig. 2A.

**Table 2:** Radial tilt in successive follow-up

RT	FU I				FU-II		FU-III		FU-IV	
				
	Pre red		Post red		At 1 month		At 3 months		At 6 months	
					
	DF	PF	DF	PF	DF	PF	DF	PF	DF	PF
<13	9	9	1	0	2	5	2	9	7	16
13-33	25	21	33	30	32	25	32	21	27	14
>33	0	0	0	0	0	0	0	0	0	0
Total	34	30	34	30	34	30	34	30	34	30

##### Palmar tilt:

At six months 23 patients (67.64%) of DF immobilized group had 1 to 21° palmar tilt as compared to nine patients (30%) in the PF immobilized group (*P* value = 0.0022). The correction achieved in palmar tilt was also lost during subsequent segmental follow-up more in the PF group in comparison to the DF group (Table 3, Fig. 2A, Fig. 2B).

**Table 3:** Palmar tilt in successive follow-up

RT	FU I				FU-II		FU-III		FU-IV	
				
	Pre red		Post red		At 1 month		At 3 months		At 6 months	
					
	DF	PF	DF	PF	DF	PF	DF	PF	DF	PF
<11	16	14	9	4	10	6	9	12	10	19
11-21	18	16	25	26	24	24	25	18	24	11
>21	0	0	0	0	0	0	0	0	0	0
Total	34	30	34	30	34	30	34	30	34	30

##### Ulnar variance:

At six months 22 patients (64.70%) in the DF group had normal variance i.e. -2 to 0 mm. In the PF group only 12 patients (40%) had normal ulnar variance (*P* value = 0.029) (Table 4).

**Table 4:** Ulnar variance in successive follow-up

UV in min	FU I				FU-II		FU-III		FU-IV	
				
	Pre red		Post red		At 1 month		At 3 months		At 6 months	
					
	DF	PF	DF	PF	DF	PF	DF	PF	DF	PF
<-2	3	4	6	5	6	3	5	1	3	0
−2 to +2	22	19	27	23	27	22	25	15	24	15
>+2	9	7	1	2	1	5	4	14	7	15
Total	34	30	34	30	34	30	34	30	34	30

#### Range of movements

##### Dorsiflexion:

A greater number of patients in the DF group showed faster improvement as compared to the PF group. At six months all 34 patients (100%) in the DF group had dorsiflexion more than 45° as compared to 13 patients (43.33%) in the palmarflexion group (*P* value = 0).

##### Palmar flexion:

At the first follow-up, at the time of plaster removal, palmarflexion in the two groups was comparable, however, at final follow-up all 34 patients (100%) of the DF group had palmarflexion more than 30° as compared to 19 patients (63.33 %) in the PF group (*P* value = 0.000073).

##### Supination:

Thirty patients (88.23%) had more than 50° supination in the DF group as compared to 21 patients (70%) in the PF group (*P* value = 0.05).

##### Pronation:

33 patients (97.05%) in the DF group had more than 50° pronation as compared to 27 patients (90%) in the PF group (*P* value = 0.217).

##### Ulnar deviation:

Thirty-three patients (97.5%) in the DF group had more than 15° ulnar deviation as compared to 21 patients (70%) in the PF group (*P* value = 0.00321).

##### Radial deviation:

Twenty-eight patients (82.35%) in the DF group had more than 15° ulnar deviation as compared to 16 patients (53.33%) in the PF group (*P* value = 0.0099).

#### Grip strength

It was measured in both dominant and non-dominant hand and scoring was done accordingly in the final follow-up. There were 26 patients (76.47%) in the DF group with more than two third grip recovery of normal side as compared to only seven patients (23.33 %) in the PF group (*P* value = 0.00002).

##### Arthritis changes

They were not seen in any of the cases in both the PF as well as DF group as the follow is very short.

##### Complications

They were seen only in the PF group where one patient presented with stiff finger in first follow-up and two more presented with stiffness in next follow-up. However, none of the patients in either group showed any complication at final follow-up.

###### End result:

At the final follow-up, 31(91.7%) patients in the DF group showed excellent to good results (Table 5) as compared to 20 (66.6%) patients in the PF group (*P* value = 0.013)

**Table 5:** Functional outcome at final follow-up according to Saito's scoring system 8

	Six months	
	
	DF	PF
Excellent (0-3)	24	12
Good (4-9)	7	8
Fair (10-15)	2	7
Poor (16-26)	1	3
Total	34	30

## DISCUSSION

No clear consensus exists as to the best position for immobilizing the wrist in a cast in extraarticular fracture of lower end radius.^13^ Sarmentio *et al.*,^17^,^18^ advocated immobilization in the position of supination to decrease the deforming force of the brachioradialis, which may cause loss of reduction. In contrast, Wahlstrom^19^ recommends immobilization in pronation because he claims that the pronator quadratus causes the deforming force and is responsible for loss of reduction.

According to the John Charnley,^20^ Colles' fracture should be treated in palmar flexion and ulnar deviation as dorsal periosteal hinge provides stability. Following this, traditionally, extra-articular fractures of the lower end of radius were classically treated by closed reduction, cast immobilization in palmar flexion and ulnar deviation. But this conventional position has higher chance of redisplacement, inhibits hand functions and has greater associated complications like median nerve compression.^10^

Van der Linden^9^ conducted a study by applying cast in different positions of wrist and compared between complete cast and splint. He studied the anatomical and functional outcome and found that the results were surprisingly same; thereby concluding that the technique of immobilization plays a subordinate role.

The concept of our study was influenced by the original recommendation by Zuppinger^10^,^21^ in 1910 and Bohler in 1929^10^,^21^ that the position of the wrist should be changed from slight palmar flexion at initial post reduction to neutral or slight extension but maintaining ulnar deviation at 10 to 14 days post reduction.

Our study resembles to some extent the study done by Gupta A^22^ in 1991 on 204 patients in which displaced Colles’ fractures were subjected to closed reduction and plaster immobilization randomly allocated to one of the three groups with respect to wrist position: Palmar flexion, neutral or dorsiflexion. They reported that in displaced extra-articular fractures with no comminution the position of the wrist made no significant difference in regards to later displacement. In comminuted fractures, both extra-articular and intra-articular, the best anatomical results were in fractures treated in dorsiflexion. Functional results in all fractures, regardless of the classification were superior if the fractures were treated in dorsiflexion.

In this study we compared the functional and radiological results of extra-articular fractures of lower end radius treated conservatively in two groups, one with wrist immobilized in DF and the other in PF, we found that individual movements of DF, PF, supination, pronation, ulnar and radial deviation as well as total range of movements are significantly better when the wrist is immobilized in DF as concluded by Gupta A.^22^ Further, grip strength recovery and subjective assessment of pain, disability and limitation of the movements was also better as well as faster in DF immobilized patients.

Radiological parameters as measured by ulnar variance, palmar tilt and radial tilt were not seen to differ much at the first follow-up between DF and PF wrist immobilized patients. However, the maintenance of the parameters in successive follow-up was found to be markedly better in the DF group as compared to the PF group. The residual deformity seemed to be greater in the PF group. Thus radial tilt, palmar tilt and ulnar variance all were found to be significantly well maintained in the DF immobilized group as compared to the PF group throughout the follow-up stating that the latter has increased chance of redisplacement.

Although arthritic changes were not seen in any of the groups possibly in view of very short followup. Complications like stiffness of fingers was seen only in the PF group at initial follow-ups and all recovered fully at subsequent follow-ups. The DF group never showed any complication even at subsequent follow-ups.

According to Gupta A^22^ the reasons for the better results in the DF immobilized wrist can be understood by understanding the biomechanics of the wrist joint and fracture reduction. In the PF group the dorsal carpal ligament is taut, but cannot stabilize the fracture because of its lack of attachment to the distal carpal row. Thus the deforming forces and the potential displacement of the fracture are parallel.^22^,^23^ While in DF immobilization the volar ligament is taut which has attachment to the distal as well as proximal carpal row and tends to pull the fracture anteriorly. The deforming forces act at an angle that tends to reduce the displacement of the fracture thus preventing redisplacement.^22^,^23^

Since the wrist in extension is the optimal position for hand function and rehabilitation of the fingers, along with the fact that PF is associated with a higher rate of fracture displacement, Gupta concluded that flexion at the fracture site is important to make use of the dorsal periosteral hinge but the flexed position need not be maintained at the wrist joint.

It is concluded that better results in DF immobilized wrist are because DF is needed for the rehabilitation of fingers, and the optimal functional position for the hand is wrist in extension. Thus in conservatively treated extra-articular fractures of the lower end radius, flexion should be at fracture site to make use of the periosteal hinge but the wrist should be immobilized in position of slight extension.^16^,^21^

**Figure 3:**
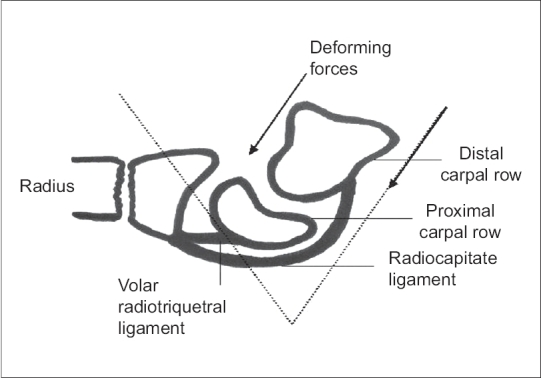
Diagramme of dorsiflexion

**Figure 4:**
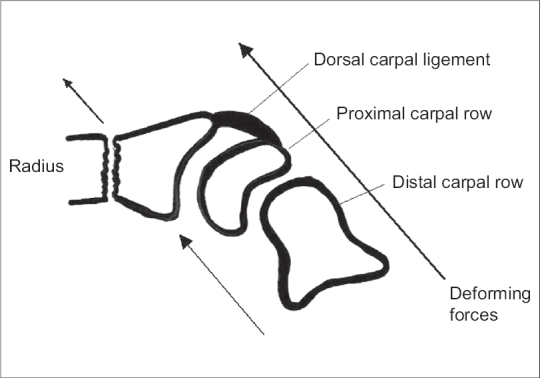
Diagramme of palmar flexion
